# Penetration and Perforation of Terminal Ileum Diverticulitis

**DOI:** 10.1155/2020/7361389

**Published:** 2020-10-27

**Authors:** Fumito Saijo, Kentaro Sawada, Ryohei Nomura, Mitsuhisa Mutoh, Yoichi Narushima, Noriyuki Iwama, Fumie Nakayama, Hiromi Tokumura

**Affiliations:** ^1^Department of Surgery, Tohoku Rosai Hospital, 4-3-21, Dainohara, Aobaku, Sendai, Miyagi 981-8563, Japan; ^2^Department of Pathology, Tohoku Rosai Hospital, 4-3-21, Dainohara, Aobaku, Sendai, Miyagi 981-8563, Japan

## Abstract

**Background:**

Terminal ileum diverticulitis is a rare clinical disease. It can frequently mimic other processes, such as acute appendicitis. Diagnosis and therapeutic decision making (surgical or conservative treatment) can be complex. We report four interesting cases of terminal ileum diverticulitis. *Case Presentation*. Case 1: a 55-year-old male presented to us with a 3-day history of severe right lower quadrant pain. Computed tomography (CT) showed penetration of terminal ileum diverticulitis. Following a 7-day conservative treatment, he underwent ileocecal resection. Pathology results revealed a false diverticulum and two in five points of perforated terminal ileum diverticulum. Case 2: a 77-year-old male presented to us with severe right lower quadrant pain and unconsciousness. CT showed penetration of terminal ileum diverticulitis and air in the mesentery. Ileocecal resection was performed 2 days postadmission. Pathology results revealed a false diverticulum and penetrated terminal ileum diverticulum. Case 3: a 61-year-old male presented to us with a right lower quadrant pain for 10 days and fever for 6 days. CT showed penetration of terminal ileum diverticulitis and abscess of the psoas muscle. Puncture and drainage of abscess were performed. Laparoscopic ileocecal resection was performed 30 days postadmission. Pathology results revealed a false diverticulum and penetrated terminal ileum diverticulum. Case 4: a 39-year-old female presented to us with right lower quadrant pain for 9 days, suspicious of appendicitis. CT showed abscess of pericecal area. Puncture and drainage were performed. A drainage tube was located into the cecum through the terminal ileum. Conservative therapy was effective, and she was discharged 23 days postadmission.

**Conclusions:**

All four cases had right lower quadrant pain. Three cases were diagnosed by CT, whereas one was diagnosed by abscess drainage. Two cases required surgical treatment within 3 days, one within about 1 month, and one case did not require surgery. The decision of whether to manage a patient surgically or conservatively is difficult. It is critical not to delay the decision of performing a surgical treatment until each patient reaches a stable general condition.

## 1. Background

Terminal ileal diverticulitis is a very rare type of ileum diverticulitis, with the exception of Meckel's diverticulum. An important characteristic of these diverticula is that they are false diverticula. Most patients with terminal ileum diverticulosis are asymptomatic. When the diverticulosis becomes diverticulitis, patients may present with right lower quadrant pain and a clinical picture that leads to suspicion of acute appendicitis. Computed tomography (CT) is very useful for the diagnosis of terminal ileum diverticulitis. However, because of abscess formation, diagnosis may be very difficult. To date, a standardized treatment has not been established in diverticulitis patients. Conservative treatment (e.g., with antibiotics and abscess drainage) may be useful at times. However, surgical treatment may be necessary in case of an unstable patient. Here, we present four cases of terminal ileum diverticulitis. For these patients, both surgical and conservative treatments have been used.

## 2. Case Presentation

### 2.1. Case 1

**Figure 1:**
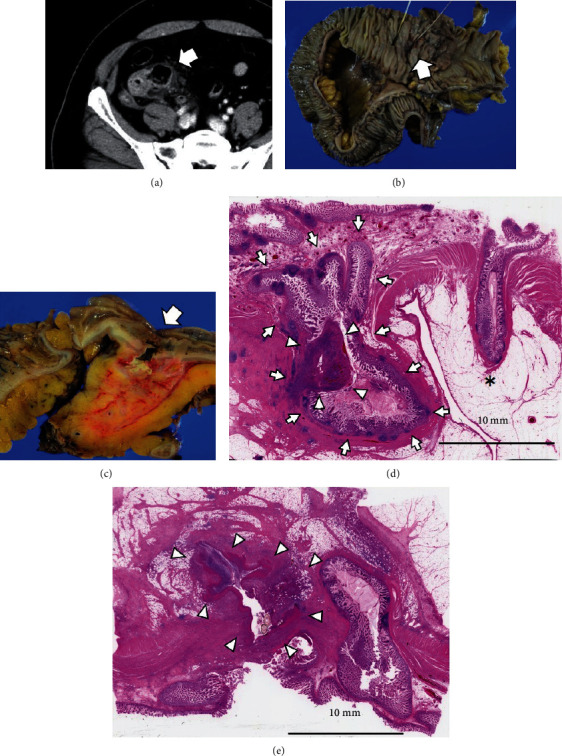
Case 1 findings. (a) Contrast-enhanced CT revealed the inflammation of mesenterium caused by penetration of a terminal ileac 2-cm diverticulitis (arrow). The CT detected air in the terminal ileum's mesentery. However, no free air was detected in the abdominal cavity. (b) Resected specimen postileocecal resection revealed at least two diverticulum. Two surgical bougie probes show diverticulum of specimens. (c) Specimen cross section of penetration from diverticulitis (arrow). (d) Histological (H&E) evaluation of the diverticulum mucosa. Inflammatory granuloma (arrowheads) was observed in the diverticulitis (arrow). An additional diverticulum was detected (asterisk). (e) Histologically (H&E), we observed severe inflammatory findings (arrowheads).

A 55-year-old male presented to the gastrointestinal department of our hospital with severe right lower quadrant pain for 3 days. Laboratory data showed inflammation, with a white blood cell (WBC) count of 17,900/*μ*L and C-reactive protein (CRP) of 18.06 mg/dL. Contrast-enhanced CT revealed the inflammation of mesenterium caused by penetration of a terminal ileum 2-cm diverticulitis. These were at least two parts of diverticulum. Additionally, the CT detected air in the terminal ileum's mesentery, whereas no free air was found in the abdominal cavity (Figure 1(a)). A 7-day conservative therapy in the form of antibiotics, levoflox 500 mg/day, was prescribed to the patient. However, his inflammation status did not improve. Consequently, an ileocecal resection as surgical therapy was performed after 7 days of antibiotic therapy. Two diverticulum at the terminal ileum were detected, along with inflammatory thickening of the mesentery (Figures 1(b) and 1(c)). Of note, 40 cm terminal ileum adhered with diverticulum and retroperitoneum. Histopathological results revealed that the diverticulum was a false diverticulum. Additionally, it found two in five points of terminal ileum diverticulum perforation (Figures 1(d) and 1(e)). The patient was hospitalized for 12 days postsurgery.

### 2.2. Case 2

**Figure 2:**
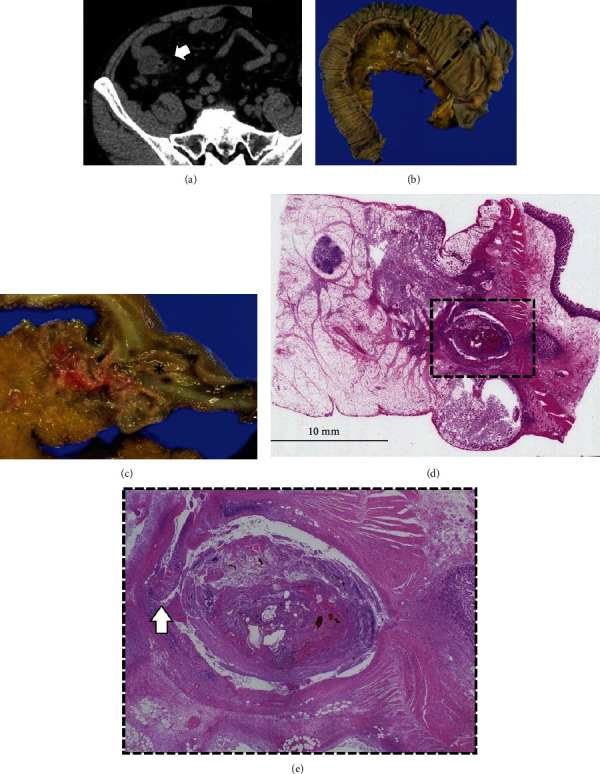
Case 2 findings. (a) CT scan showed penetration of terminal ileum diverticulitis and air (white arrow) in the near mesentery. (b) Resected specimen after ileocecal resection showed the presence of diverticulum. The dotted line represents the cross section line for Figure 2(c). (c) Specimen cross section of penetration from diverticulitis (asterisk). (d) Microscopic feature (H&E). Inflammation due to diverticulitis is observed in the area enclosed by the dotted square. (e) Microscopic examination (H&E) of the tissue within the dotted square from Figure 2(d). An area of inflammation was observed in the intestinal mucosa (arrow) with diverticulitis.

A 71-year-old male presented to the emergency department of our hospital with severe right lower quadrant pain and unconsciousness. Laboratory data showed inflammation status, with a WBC count of 13,800/*μ*L and CRP of 8.82 mg/dL. CT showed penetration of terminal ileum diverticulitis and air in the near mesentery (Figure 2(a)). The patient was surgically treated with an ileocecal resection 2 days postadmission (Figures 2(b) and 2(c)). An abscess at the mesentery of the terminal ileum was identified and covered by ileum wall. Pathological examination revealed that the diverticulum was a false diverticulum and penetrated terminal ileum diverticulum (Figures 2(d) and 2(e)). The patient was hospitalized for 10 days postsurgery.

### 2.3. Case 3

**Figure 3:**
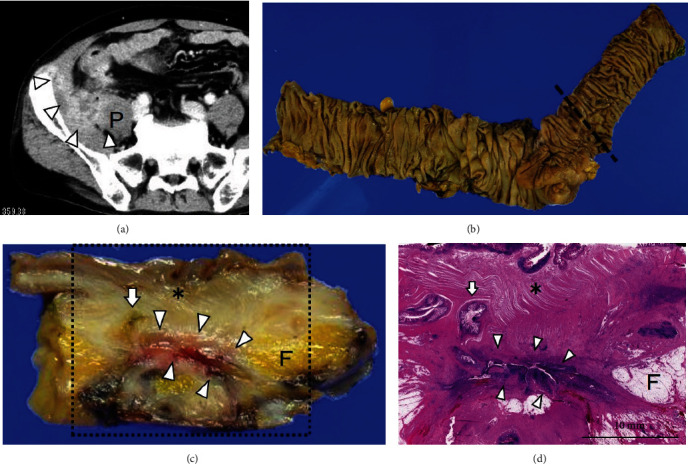
Case 3 findings. (a) CT showed abscess formation by penetration of terminal ileum diverticulitis (arrowheads) into the psoas muscle (P) and retroperitoneum. (b) Resected specimen after ileocecal resection showed the presence of diverticulum. The dotted line represents the cross section line for Figure 3(c). (c, d) Specimen cross section and microscopic examination (H&E) shows terminal ileum diverticulum (asterisk), intestinal mucosa (arrow), inflammation area, and intestinal mucosa (arrowheads), and fibrosis area (F).

A 61-year-old male presented to the surgical department of our hospital with right lower quadrant pain for 10 days and fever for 6 days. Prior to visiting our hospital, an oral antibiotic therapy was initiated in another hospital. Laboratory data showed an inflammation state, with a WBC count of 13,200/*μ*L and CRP of 10.05 mg/dL. CT showed penetration of the terminal ileum diverticulitis and an abscess from the terminal ileum through the psoas muscle until the inguinal area (Figure 3(a)). Puncture and drainage abscess were performed. Laparoscopic ileocecal resection was performed 30 days postadmission (Figures 3(b) and 3(c)). Of note, 10 cm of the terminal ileum adhered with the retroperitoneum and right abdominal wall. Pus was found during adhesiolysis of the terminal ileum. Histopathological results revealed that the diverticulum was a false diverticulum and showed penetrated terminal ileum diverticulum (Figure 3(d)). The patient was hospitalized for 11 days postsurgery.

### 2.4. Case 4

**Figure 4:**
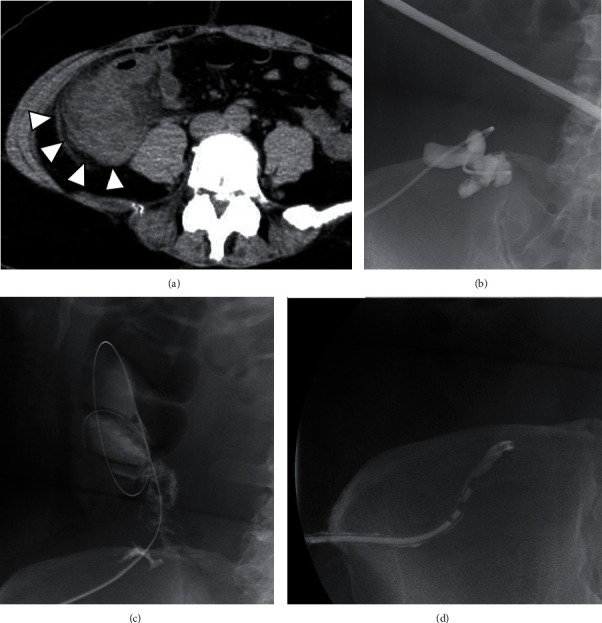
Case 4 findings. (a) CT showed abscess formation by penetration of terminal ileum diverticulitis (arrowheads). (b) Fluoroscopy examination showed the abscess puncture and drainage using 7Fr PTC Drainage tube (Pigtail type, CREATE MEDIC CO., LTD., Japan) were performed. At this time, the ileum and cecum were not visualized. (c) Fluoroscopy examination showed the presence of guidewire and drainage tube inside the cecum through the terminal ileum after 5 days of drainage. (d) Fistography examination using a drainage tube did not show terminal ileum after 13 days.

A 39-year-old female presented to the emergency department of our hospital with right lower quadrant pain for 9 days. Appendicitis was therefore suspected. Prior to visiting our hospital, oral antibiotic therapy had been initiated in a different hospital. Laboratory data showed an inflammation state, with a WBC count of 16,500/*μ*L and CRP of 22.01 mg/dL. CT showed a 5 cm abscess of the pericecal area (Figure 4(a)). Puncture and drainage using a 7Fr PTC Drainage tube (Pigtail type, CREATE MEDIC CO., LTD., Japan) were performed (Figure 4(b)). Drainage tube existed within the cecum through the terminal ileum after 5 days of drainage (Figure 4(c)). At that time, a diagnosis of terminal ileum diverticulum perforation was made. The drainage catheter was changed into a 10Fr PTC Drainage tube (Straight type, CREATE MEDIC CO., LTD., Japan) (Figure 4(c)). Fistography examination using the drainage tube did not show the terminal ileum after 13 days (Figure 4(d)). A fistula from the terminal ileum diverticulum was closed. For this patient, conservative therapy was effective, and the patient was discharged 23 days postadmission.

## 3. Conclusions

Small bowel diverticulosis is mostly an asymptomatic rare disease. Its incidence varies from 0.3% to 2.3% in the general population [1, 2]. Diverticulitis disease is more common in the proximal jejunum (75%), followed by the distal jejunum (20%), and the ileum (5%) [3]. Small intestine diverticulum affects men more frequently than women. It manifests itself mostly in 60–70-year-old individuals [4]. The etiology of the small intestine diverticulum is mostly unknown. However, it is thought that motility disorders of the small intestine, local fragility of the smooth muscle, and a partial rise of intestinal pressure are involved [5, 6]. In contrast to true congenital Meckel's diverticulum, false diverticulum is usually multiple and occurs at the mesenteric border [7]. Of the cases presented here, as shown by the pathological findings, the diverticulum of Cases 1–3 involves the mesenteric border, multiple diverticulum, and penetration into the mesentery.

Ultrasound and CT are very useful examination for a differential diagnosis between appendicitis, ileum diverticulum, Crohn's disease, and other diseases [1]. The four cases presented here were characterized by the same symptoms of right lower quadrant pain. Of note, three cases were diagnosed by CT and one by abscess drainage.

This condition can often mimic other processes, such as acute appendicitis. In case appendicitis had caused a local abscess around the right lower abdomen, conservative therapy (i.e., antibiotics or abscess drainage) could be the treatment of choice, given a stable general condition of the patient. Two cases required surgical therapy within 3 days, one case within about 1 month, and one case did not need surgery. Several earlier reports suggested that a conservative management may be sufficient to treat uncomplicated terminal ileum diverticulitis without perforation [8, 9]. However, early surgical treatment represents the approach of choice for patients with confirmed diverticulitis and serious complications. Of note, a delayed diagnosis of this clinical entity has a high morbidity and mortality rate [10]. All the cases presented here were perforated or had a penetrated status. As a consequence, the choice between surgical or conservative therapy was difficult. It is critical not to delay the decision of performing a surgical treatment until each patient reaches a stable general condition.

Surgical treatment was necessary for three cases. Specifically, one was performed laparoscopically and two with an open approach. Of note, of the surgically treated cases, an acute inflammation status was observed in the two open approach cases, and a chronic status was found in the laparoscopic case. Specifically, Case 3 had an abscess from the terminal ileum through the psoas muscle until the inguinal area. Although surgical therapy may be required, a stable inflammation status would be of preference. Laparoscopic surgery was performed only in Case 3. Cases 1 and 2 might be possible by laparoscopic approach, but the operator in charge chose open surgery because of its skill and safety.

One case underwent drainage therapy. If the patient reached a stable status only by drainage, it would be acceptable. In that case, no surgical operation was required. In general, surgical therapy faces the possibility of complications both during and after surgery. This case underwent a safe drainage and experienced good progress. However, future diverticulitis recurrence is possible. Similarly, bowel stenosis may develop after repeated diverticulitis. As a consequence, patients should receive follow-up.

We experimented four cases of terminal ileum diverticulitis perforation and penetration. Terminal ileum diverticulitis mimicked appendicitis. CT was a useful tool for diagnosis. Surgical treatment or abscess drainage may be required until the patient reaches a stable status. Laparoscopic surgery will be technically safe and feasible where expertise of laparoscopy is available. The precise method for the surgeon to determine is safe should be selected.

## Abbreviations

WBC: White blood cell
CRP: C-reactive protein
CT: Computed tomography.
